# Supplementary material for: Common noctules exploit low levels of the aerosphere
Source: R Soc Open Sci. 2019 Feb 27;6(2):181942. doi: 10.1098/rsos.181942 (PMC6408413; doi:10.1098/rsos.181942)
Supplement: Supplementary Figures [file rsos181942supp1.pdf]

Supplemental information for O'Mara et al: **Common noctules exploit low levels of the aerosphere**

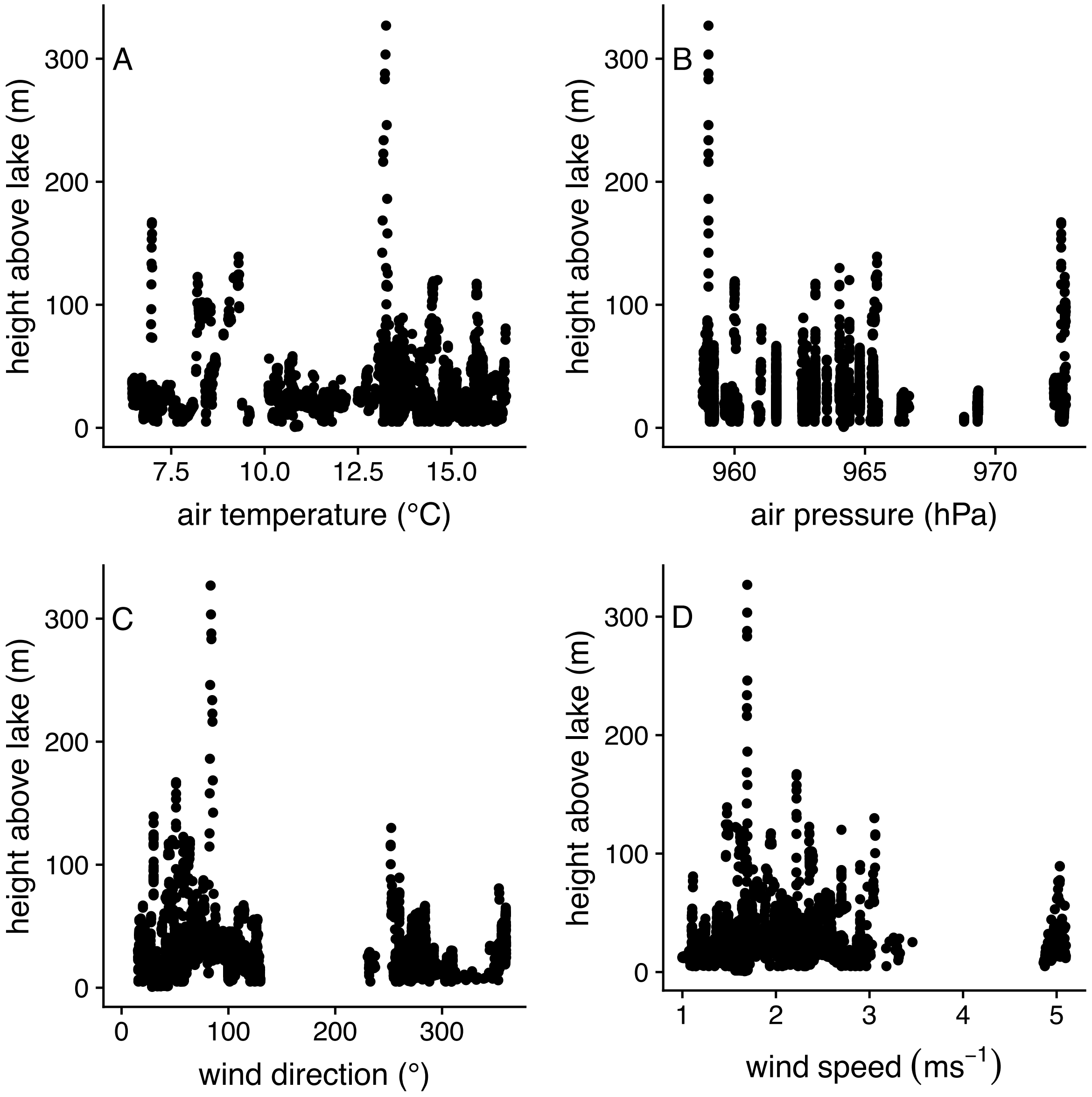

Figure S1. Flight heights above Lake Constance and the corresponding A) air temperature, B) air pressure, C) wind direction, and D) wind speed.

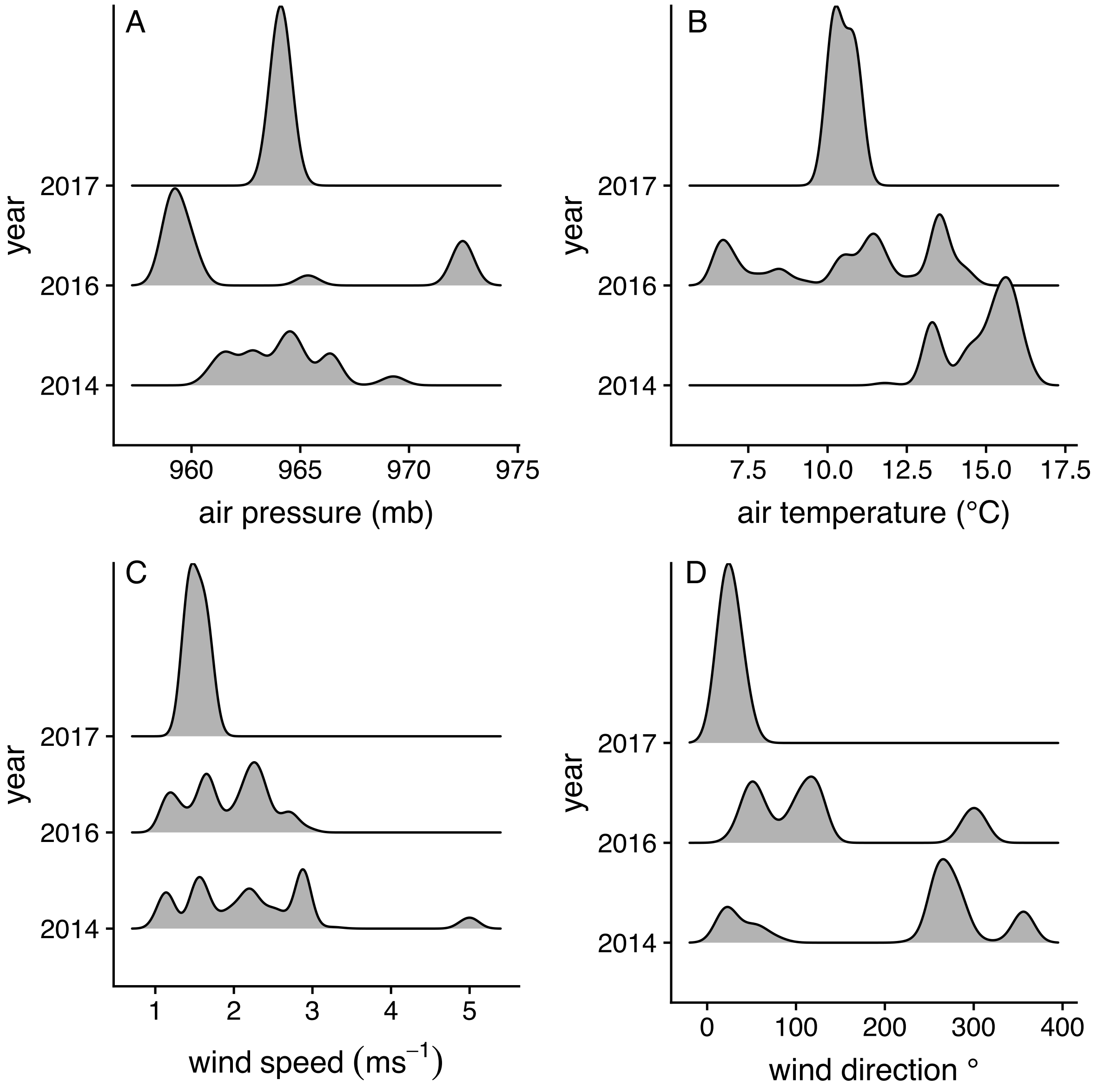

Figure S2. Yearly density plots for A) air pressure, B) air temperature, D) wind speed, and D) meteorological wind direction during noctule height tracking. Densities are calculated from the time-interpolated samples calculated between hourly measures from the Konstanz weather station.
